# The cost of banning TikTok: Implications for the digital advertising market

**DOI:** 10.1073/pnas.2512043122

**Published:** 2025-09-15

**Authors:** Dante Donati, Hortense Fong

**Affiliations:** ^a^Marketing Division, Columbia Business School, Columbia University, New York, NY 10027

**Keywords:** digital advertising, market concentration, ad prices, digital regulation

## Abstract

Social media platforms have become vital channels for businesses to reach consumers through advertising. But in the United States, the digital advertising market in which these platforms operate is dominated by a few major players, raising concerns for antitrust regulators. In such a concentrated market, the entry or exit of a single platform can reallocate billions in ad spending, affecting businesses and users. TikTok’s temporary suspension in the United States in January 2025 provides a unique natural experiment to examine how the removal of a major player would shift advertising demand and supply on competitors, specifically Facebook and Instagram, revealing the degree of substitutability across platforms and the intensity of competition. Using a difference-in-differences approach comparing advertising activity in the United States to other countries, we find that Meta ad volume and spend rose by 6.3% and 22.4%, as a result of the outage, without a corresponding increase in ad impressions. Consequently, Meta ad prices, as measured by cost per thousand impressions, jumped by 12.1%. Shifts in demand were three times greater for larger advertisers relative to smaller ones, suggesting that Meta platforms and TikTok are closer substitutes for larger firms and that a TikTok ban would therefore impose greater challenges on smaller businesses.

With 73% of Americans using social media, platforms like TikTok, Instagram, Facebook, and YouTube have become vital channels for businesses to reach consumers. These platforms are where millions of businesses advertise to build visibility and sell products. In the highly concentrated U.S. digital advertising market, where Meta and Google account for nearly half of all digital ad revenue (1), disruption to any of these platforms can significantly alter the ecosystem in which businesses advertise and users engage.

Social media platforms provide a marketplace for advertisers to bid on user attention. They convert user traffic into ad impression opportunities, balancing ad load and organic content to optimize user experience and revenue. Demand comes from advertisers, who set campaign budgets and compete for impressions through auctions. The core pricing metric is the cost per thousand impressions (CPM), which reflects the price of showing an ad 1,000 times.

Event-study estimates of the effect of the TikTok outage on ad spend on Meta (*A*), CPM on Meta (*B*), and ad spend on TikTok (*C*). Panels (*A* and *B*) use advertiser-day data for SIEP advertisers. Panel (*C*) uses country-day data for all TikTok advertisers. The lines report regression coefficients on US-by-day interactions, using Jan 18 as baseline. Panel (*A*) uses log(spend+1). Panels (*A* and *B*) include advertiser, country, and day fixed effects, and past expenditure, USD, and language indicators interacted with a postoutage indicator. Panel (*C*) includes country and day fixed effects. SEs are clustered at the country level. Bars show 95% CIs. “SIEP advertisers on TikTok” refers to SIEP advertisers in the United States identified as having advertised on TikTok in the year preoutage.

Currently, TikTok faces a potential U.S. ban unless divested from its Chinese parent company. Such a ban would further increase market concentration, raising concerns for antitrust regulators and businesses that advertise on social media (2). A key question for regulators is how the removal of a major player like TikTok would shift advertising demand and supply, revealing the degree of substitutability across platforms and the intensity of competition. Regulators value competition because it typically leads to lower prices, higher quality, and greater innovation. The TikTok outage on January 19, 2025, in the United States provides a natural experiment to quantify short-term shifts in advertising demand on other social media platforms.

Furthermore, consistent with the effects of other ad tech regulation (3, 4), we expect a TikTok ban to be more harmful to smaller businesses than to larger ones. Smaller advertisers often face greater constraints, such as limited expertise, experience, and budgets, making switching to other platforms more challenging (5). Additionally, TikTok estimated that a ban could cost small businesses over $1B in monthly revenue, as the platform has been particularly valuable in helping them gain visibility and engage with targeted audiences (6). We therefore examine how larger versus smaller businesses adjusted their advertising spending, helping us understand how a TikTok ban could differentially impact them.

Finally, a key question for advertisers is how a TikTok ban would affect ad prices on other platforms. In theory, the effect is ambiguous. TikTok’s 170 million monthly U.S. users, averaging 53.8 daily minutes, would likely shift to other platforms like Facebook and Instagram, increasing the supply of ad impressions and lowering prices (7–9). Meanwhile, TikTok’s $11 billion in annual ad spend would shift to rivals, with Meta’s platforms likely gaining 40% (10). Higher demand would increase ad prices (11). Ultimately, prices will reflect these relative shifts in supply and demand.

Heterogeneous effects of the TikTok outage by advertiser size on Meta ad spend (*A* and *B*), and TikTok ad spend (*C*). Panels (*A* and *B*) use log(spend+1) and advertiser-day data for SIEP advertisers. Panel (*B*) includes only advertisers active on Meta on January 19. In panels (*A* and *B*), advertisers are grouped based on total Meta ad spend in the two months prior to the outage: smaller (below the country-specific median) and larger (above the median). Panel (*C*) uses country-day data for all advertisers on TikTok, with advertiser size based on TikTok’s internal classification (*SI Appendix*). Lines show regression coefficients on US-by-day interactions. The reference day is January 18 for (*A* and *C*), and January 19 for (*B*). Regressions in (*A* and *B*) include advertiser, country, and day fixed effects; regressions in (*C*) include country and day fixed effects. SEs are clustered at the country level. Bars indicate 95% CIs.

## The TikTok Outage as a Natural Experiment

We leverage TikTok’s outage in the United States as a quasi-exogenous shock to advertising demand and supply on Meta platforms. On January 19—the day the ban was scheduled to take effect—TikTok went dark for 14 h before coming back online after the ban was delayed. During the outage, content was inaccessible, and even after TikTok returned, service restoration was slow, resulting in minimal traffic that day and reduced traffic the following week.

To study the substitutability between TikTok and Meta, we collect publicly available data from the Meta Ad Library for thousands of advertisers across multiple ad categories, including Social Issues, Elections, or Politics (SIEP), Housing, Employment, and Financial Products and Services. While all categories provide information on the timing and number of ads, only the SIEP category provides ad spend and impressions, enabling us to study shifts in budget allocation and prices. We interpret ad spend as a proxy for demand, as it typically reflects the advertiser’s budget set when a campaign is launched. In the week before the outage, 47% of SIEP advertisers had an active ad on any given day, with an average daily spend of $140 and a CPM of $9.1. Meanwhile, 72% of non-SIEP advertisers had an active ad on any given day. Additional descriptive statistics are in *SI Appendix*.

We estimate a difference-in-differences (DiD) model comparing the United States to 32 other countries before and after the outage, for advertisers running ads in the two weeks around the event. The other countries serve as a natural control group, as TikTok’s availability remained unchanged outside the United States. Although TikTok prohibits political ads, the set of SIEP ads is broader, allowing us to document potential substitution effects.^*^ To directly quantify substitution, we obtain from TikTok—through fuzzy matching on page name—which U.S. advertisers in our SIEP sample advertised on TikTok during the previous year (*SI Appendix*), and find that 12% of the SIEP advertisers had. Additional data from the non-SIEP categories on advertising activity and volume strengthen the robustness of our analysis. Last, we use proprietary TikTok country-day-level data on spend to study advertising activity on the platform after it returned online.

## Ad Spend, Prices, and Impressions on Meta

On the day of the outage (January 19), SIEP ads experienced a 5.4 percentage point (p.p.) rise in launch likelihood, a 6.3% increase in ad count, and a 22.4% boost in spend on Meta (Table 1 columns 1 to 3). Effects were approximately 50% larger for advertisers that had previously run ads on TikTok, capturing direct substitution to Meta (columns 4 to 6).

**Table 1.** Advertisers’ demand for Meta ads, prices, and impressions on the day of the TikTok outage

	Demand for ads									
	SIEP advertisers			SIEP advertisers on TikTok			Non-SIEP advertisers		Metrics of active SIEP ads	
	(1) Any ad (1/0)	(2) (log) No. ads	(3) (log) Spend	(4) Any ad (1/0)	(5) (log) No. ads	(6) (log) Spend	(7) Any ad (1/0)	(8) (log) No. ads	(9) (log) CPM	(10) (log) Impressions
US × Outage	0.054^∗∗∗^ (0.019)	0.063^∗∗^ (0.030)	0.224^∗∗∗^ (0.076)	0.083^∗∗∗^ (0.019)	0.095^∗∗∗^ (0.032)	0.321^∗∗∗^ (0.075)	0.058^∗∗∗^ (0.013)	0.087^∗∗∗^ (0.014)	0.121^∗∗^ (0.051)	−0.011 (0.048)
FE & Controls	✓	✓	✓	✓	✓	✓	✓	✓	✓	✓
Observations	238,428	238,428	238,428	204,005	204,005	204,005	787,236	787,236	108,730	108,730
No. of advertisers	29,708	29,708	29,708	25,432	25,432	25,432	90,589	90,589	19,278	19,278
Adj. R-squared	0.593	0.846	0.761	0.578	0.829	0.745	0.656	0.854	0.974	0.942
Baseline US mean	0.49	6.42	233.15	0.53	9.08	301.10	0.83	3.44	20.86	27,623.98

Each observation is an advertiser-day. The panel includes observations between Sun, Jan 12, and Sun, Jan 19, for advertisers running at least one ad campaign between Jan 12 and 26. Columns (1 to 3) refer to all SIEP campaigns. “SIEP advertisers on TikTok” (columns 4 to 6) refers to SIEP advertisers in the United States identified as having advertised on TikTok in the year prior to the outage. Columns (7 to 8) refer to non-SIEP ad categories: housing, employment, and financial products and services. For the continuous variables in columns (1 to 8), we use log(y+1). Columns (9 to 10) include only active campaigns, i.e., those with positive ad spend. Outage = 1 on the day of the outage (Jan 19), US = 1 if the advertiser ran ads in the United States. SEs are heteroskedasticity-robust and clustered at the country level. Separate fixed effects for advertiser, country, and day are included. Controls in columns (1 to 6 and 9 to 10) include past expenditure, USD currency, and language indicators interacted with Outage. Controls in columns (7 to 8) include ad category and language indicators interacted with Outage. ^∗^(P<0.1), ^∗∗^ (P<0.05), ^∗∗∗^ (P<0.01).

A potential concern is that the January 20 U.S. presidential inauguration may have driven some of the SIEP ad surge, given possible event-related advertising content. We therefore run the same DiD analysis with non-SIEP ads, which are unlikely to suffer from this concern. We observe comparable effects with a 5.8 p.p. rise in launch likelihood and an 8.7% increase in ad count (columns 7 to 8). Additional analyses in *SI Appendix* of the SIEP data show no evidence that the inauguration drove the demand increase.

Turning to prices for SIEP ads, we find average CPM increased 12.1% (column 9), driven by higher demand without a matching rise in impressions (column 10).^†^ A series of placebo tests and robustness checks across model specifications (*SI Appendix*) support our findings and identification strategy.

**Fig. 1.**
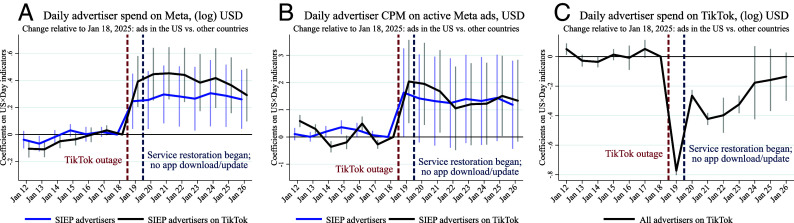
Event-study estimates of the effect of the TikTok outage on ad spend on Meta (*A*), CPM on Meta (*B*), and ad spend on TikTok (*C*). Panels (*A* and *B*) use advertiser-day data for SIEP advertisers. Panel (*C*) uses country-day data for all TikTok advertisers. The lines report regression coefficients on US-by-day interactions, using Jan 18 as baseline. Panel (*A*) uses log(spend+1). Panels (*A* and *B*) include advertiser, country, and day fixed effects, and past expenditure, USD, and language indicators interacted with a postoutage indicator. Panel (*C*) includes country and day fixed effects. SEs are clustered at the country level. Bars show 95% CIs. “SIEP advertisers on TikTok” refers to SIEP advertisers in the United States identified as having advertised on TikTok in the year preoutage.

Fig. 1 presents event-study coefficients for spend and CPM using January 18 as the baseline. Advertiser spend on Meta remained elevated throughout the week following the outage (*A*), as did CPM (*B*). Correspondingly, ad spend on TikTok remained lower for several days postoutage (*C*). A potential explanation for this persistence is advertisers’ uncertainty around TikTok’s future (12). While our demand estimates reflect short-run shifts, they likely understate the full extent of substitution that would occur under a permanent ban, since uncertainty would dissipate and advertisers would have more time to reallocate budgets and adjust strategies.

## Not All Advertisers Responded Equally

**Fig. 2.**
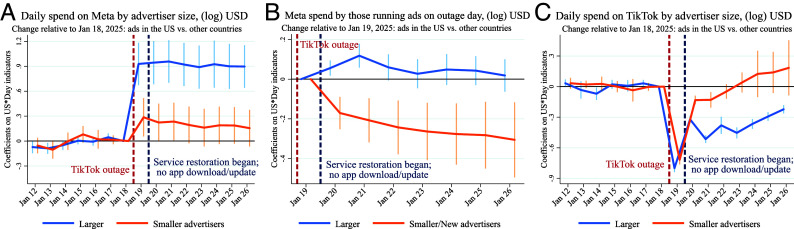
Heterogeneous effects of the TikTok outage by advertiser size on Meta ad spend (*A* and *B*), and TikTok ad spend (*C*). Panels (*A* and *B*) use log(spend+1) and advertiserday data for SIEP advertisers. Panel (*B*) includes only advertisers active on Meta on January 19. In panels (*A* and *B*), advertisers are grouped based on total Meta ad spend in the two months prior to the outage: smaller (below the country-specific median) and larger (above the median). Panel (*C*) uses country-day data for all advertisers on TikTok, with advertiser size based on TikTok’s internal classification (*SI Appendix*). Lines show regression coefficients on US-by-day interactions. The reference day is January 18 for (*A* and *C*), and January 19 for (*B*). Regressions in (*A* and *B*) include advertiser, country, and day fixed effects; regressions in (*C*) include country and day fixed effects. SEs are clustered at the country level. Bars indicate 95% CIs.

Using past expenditure to proxy business size, we analyze differences in SIEP spend estimates. On outage day, spend increased by 67% for larger advertisers, 22% for smaller advertisers (all P<0.01), and did not change for new ones. Fig. 2*A* presents the event-study estimates, confirming that the effects on spend were roughly three times greater for larger advertisers. These findings suggest that Meta platforms serve as a better substitute for them than for smaller ones. We further find that smaller advertisers who chose to advertise on Meta platforms during the outage significantly reduced their ad spend on Meta when TikTok was restored, while larger advertisers did not change their behavior (Fig. 2*B*). Without TikTok as an advertising channel, smaller advertisers would be disproportionately negatively affected by a permanent ban. Internal TikTok data reported in Fig. 2*C* confirm that spend on TikTok stayed low for larger firms but rebounded quickly for smaller firms, which highlights small businesses’ preference for TikTok as an advertising channel.

## Discussion

Our results provide important insights for policymakers and advertisers seeking to understand the unintended effects of digital regulation. First, we document a substantial shift in demand for ads from TikTok to Meta, revealing cross-platform substitution. This suggests that a TikTok ban would reduce advertisers’ options and further increase concentration in the digital advertising market. Second, removing TikTok would raise short-term ad prices, reducing advertisers’ return on ad spend. However, because remaining platforms can adjust the supply of ad slots over time, the long-term impact on prices remains unclear (11). Third, larger advertisers shifted a greater percentage of ad spend than smaller advertisers, suggesting that smaller firms, often more resource-constrained, would bear a greater burden from the cost of switching. Policymakers should weigh these unintended consequences when considering regulatory actions in the digital space.

## Materials and Methods

Using publicly available Meta Ad Library data, we constructed a dataset of ads that targeted users in the United States or 32 other countries from January 12 to 26. We aggregated the ad-level data into a panel at the advertiser-day level. The SIEP sample includes 4,920 advertisers running ads in the United States and 24,819 in the other countries. The other categories include 7,090 advertisers in the United States and 83,611 elsewhere. We supplement this with proprietary TikTok spend data at the country-day level. Additional methodological details are in *SI Appendix*.

## Supplementary Material

Appendix 01 (PDF)

## Data Availability

All publicly available data that are allowed by Meta Ad Library’s terms of service, code, and a PDF file with instructions on how to download/obtain additional data that we cannot share publicly have been deposited in Figshare (https://doi.org/10.6084/m9.figshare.29876759.v3) (13).

## Footnotes

^*^Using OpenAI’s GPT API, we estimate that one to two-thirds of U.S. SIEP ads could run on TikTok (*SI Appendix*).

^†^The price increase matches the rise in spend on active campaigns at the intensive margin (10.8%, P<0.05). The 22.4% spend increase in column (3) of Table 1 reflects the overall change.
