# Supplementary material for: The cost of banning TikTok: Implications for the digital advertising market
Source: Proc Natl Acad Sci U S A. 2025 Sep 15;122(38):e2512043122. doi: 10.1073/pnas.2512043122 (PMC12478136; doi:10.1073/pnas.2512043122)
Supplement: Supplementary file 1 — Appendix 01 (PDF) [file pnas.2512043122.sapp.pdf]

Supporting Information for

The cost of banning TikTok: implications for the digital advertising market

Dante Donati and Hortense Fong

Dante Donati.
E-mail: dd3137@gsb.columbia.edu

This PDF file includes:

1. Extended Materials
2. Extended Methods

1. Extended Materials

1.1. Replication package. We share all publicly available data that are allowed by Meta Ad Library's terms of service. For data that we cannot legally share, we provide instructions on how to collect the data. All code necessary for the replicability of our results has been made available. TikTok data are proprietary and cannot be shared. The study did not involve human subjects.

1.2. Meta Ad Library raw data. Using data from the Meta Ad Library, we construct two ad data sets. One data set includes all ads from the "Social Issues, Elections or Politics" (SIEP) category targeting users in the U.S. or 32 other countries.* The SIEP category is unique in that it provides data on advertising spend, number of impressions, estimated audience size, and audience demographics for the full universe of advertisers across many countries, including the US.† Although we are limited in the category of ads for which we can obtain data on spend and impressions, the SIEP ads still participate in the same ad auctions as other ads.‡

From the ad library, for SIEP ads we collect the following variables to use in the analysis:

- Ad ID (unique identifier for the ad)
- Page ID (identifier of the Facebook/Instagram page used to advertise)
- Country where the ad was run
- Currency used to pay for the ad
- Language of the ad
- Ad delivery start and end dates
- Placement Platform (Facebook, Instagram, Audience Network, Messenger)
- Amount Spent (range of amount spent)
- Impressions Received (range of impressions the ad received)
- Estimated Audience Size (range of estimated number of users that met targeting criteria)
- Demographic Information (age, gender, location % associated with users that met targeting criteria)

When data is provided in a range, we assign its value as the midpoint of the range. We use the currency to convert spend into USD. We drop duplicate ads (i.e., those with the same ad ID) that appear in multiple countries, as we cannot determine their targeted location unequivocally. Our data set contains 448,494 unique ad IDs across 43,525 page IDs in the SIEP category.

The second ad data set includes ads from the following specific categories identified by Meta: Housing, Employment, and Financial Products and Services. For them, we collect data via the Meta Ad Library API for a subset of countries for which these non-SIEP ads are available (these include 20 EU countries, plus the UK and Canada). For these countries, we obtain the universe of ads that were delivered to at least one user in the EU. We complement this data with a sample of ads delivered to users in the U.S. manually collected from the Meta Ad Library.

For the non-SIEP ads, we collect Ad ID, Page ID, Country, Ad delivery start and end dates, Ad Category, and Language. Our U.S. sample covers approximately 56% of all daily ads active between January 12 and 19 across the three categories, with stable coverage over time. Similar to our approach with SIEP ads, we exclude from the initial non-SIEP sample any duplicate ads that appear in multiple countries, as we cannot unequivocally determine their targeted location and, therefore, their treatment assignment. Our final non-SIEP data set contains 771,187 unique ad IDs across 95,299 page IDs.

1.3. Advertiser-level data set construction. We aggregate the ad-level data and construct panel data sets at the advertiser-day level. An advertiser is identified by a unique page ID-currency combination that can run ads in one or multiple countries.§ We focus on the period from January 12 to 26 in the main analysis to capture the weeks before and after the outage. For the SIEP ads, spend and impressions are provided for each ad over its active period. We convert these values to daily measures by equally distributing them over the active number of days. We assume a uniform distribution as we cannot observe the exact numbers each day. We test alternative distribution assumptions in Section 2.8 of the SI Appendix.

For SIEP ads, our panel contains 4,920 advertisers running ads in the US, and 24,819 doing so in the other 32 countries. There is exactly one record per advertiser-country-day and we retained observations with zero ad spend and activity, making the panel balanced. Descriptive statistics show that 47% of SIEP advertisers have an active ad on any given day in the week before the outage, and the average advertiser has 3.3 daily active ads, spends 140 USD on them, and obtains more than 30,000 daily impressions. The average CPM is 9.1 USD. Examples of U.S. advertisers include Action Against Hunger USA, DailyWire+, and World Bank Publications.

For non-SIEP ads, our panel contains 7,090 advertisers running ads in the US, and 83,611 doing so in the EU, UK, and Canada. There is exactly one record per advertiser-country-day and we retained observations with zero ad activity, making the panel balanced. Descriptive statistics show that 72% of non-SIEP advertisers have an active ad on any given day in the week before the outage, and the average advertiser has 4.2 daily active ads. Examples of U.S. housing advertisers include The Timbers at Issaquah Ridge Apartments, Indulge Real Estate, and LBI Properties. Examples of U.S. employment advertisers include Career Leap, PrideStaff, and Pivot Technology School. Examples of U.S. financial products and services advertisers include Elder CDJR Cedar Creek, Volvo Cars Rochester, and Quility Insurance.

* List of countries in the data: Australia, Austria, Belgium, Brazil, Canada, Chile, Colombia, Croatia, Denmark, Finland, France, Germany, Greece, Hungary, India, Ireland, Israel, Italy, Kosovo, Lithuania, Mexico, Netherlands, Norway, Poland, Portugal, Romania, Serbia, Slovakia, Spain, Switzerland, Turkey, United Kingdom, United States.

† https://www.facebook.com/business/help/167836590566506

‡ In the US, spending on ads categorized as "Social Issues, Elections, or Politics" accounted for about 1.65% of total Meta ad spend between 2018 and 2024, amounting to $5.24 billion out of a total $317 billion.

§ For instance, if page A operates two ad accounts that use different currencies, 1 and 2, to advertise in country c, these accounts will be assigned unique identifiers $A1, c$ and $A2, c$.

1.4. Political vs. non-political SIEP ads. TikTok does not allow political ads, and so one concern is whether the ads we see on Meta during the TikTok outage could have resulted from direct substitution. To get a sense of what proportion of ads could be shown on both TikTok and Meta platforms, we use GPT to try to predict whether each ad on Meta would be allowed by TikTok. We use two different prompts with gpt-4o-nano to try to simulate whether each ad would be allowed. The first prompt asks for GPT to determine whether each ad is political or not, and the second prompt is based on information from TikTok's political ad policy.

Prompt 1: "You are a helpful assistant that labels advertisements shown on Meta platforms based on their characteristics. This ad was shown in {country} and you have a lot of knowledge about this country. Given the following ad details, decide whether the ad is 'Political', 'Not Political', or 'Unsure'. An ad is political if it discusses elections, public office, policy, political campaigns, or candidates — including anything related to Donald Trump or his inauguration (e.g. mentions of Trump, MAGA, Inauguration Day, inaugural events, etc.) — or is by a political party, candidate, or politician. Return exactly 'Political', 'Not Political', or 'Unsure'.

- Languages: {language}
- Page Name: {page name}
- Byline: {byline}
- Ad Creative Text: {ad creative body}
- Ad Creative Link Title: {ad creative link title}

Your answer should be either 'Political', 'Not Political', or 'Unsure'."

Prompt 2: "You are an employee at TikTok's Trust & Safety team, responsible for enforcing TikTok's policy that "We do not allow political content to feature in advertising." This ad was shown in {country}, and you have deep knowledge of this policy. TikTok prohibits any ad that:

- References, promotes, or opposes candidates or nominees for public office, political parties, or government officials (including spouses or royal family members with official duties).
- Advocates for or against referenda, ballot measures, or legislative, judicial, or regulatory outcomes or processes (e.g., promoting or attacking government policies or track records).
- References elections (voter registration, turnout, appeals for votes).
- Uses campaign slogans, symbols, or logos, or sells merchandise featuring prohibited individuals/entities.

Given the following ad details, decide whether this ad is "Allowed" (complies with TikTok policy), "Not Allowed" (violates the policy), or "Unsure" (not enough information).

- Languages: {language}
- Page Name: {page name}
- Byline: {byline}
- Ad Creative Text: {ad creative body}
- Ad Creative Link Title: {ad creative link title}

Return exactly one of: Allowed, Not Allowed, or Unsure."

We use gpt-4.1-nano-2025-04-14 with a temperature of 0. The first prompt classifies roughly 1/3 of U.S. ads as political and the second prompt classifies roughly 2/3 of U.S. ads as political. Therefore, the remaining ads on Meta could potentially have come from TikTok. We are only able to classify ads and not advertisers as the different platform policies may lead to different advertising content on each platform. Because of potential measurement error in this approach, we collect additional information from TikTok to more cleanly identify direct substitution, as detailed in the next section.

1.5. Additional information from TikTok. We collect additional information from TikTok to identify a set of U.S. advertisers within our SIEP data that also previously advertised on TikTok within the last 12 months. Specifically, TikTok used fuzzy matching on the page names we provided (e.g., Action Against Hunger USA). The fuzzy matching relied on a token-based matching method, which breaks strings into tokens and compares the set of tokens for scoring. For example, using this algorithm, "Action Against Hunger USA" and "USA Action Against Hunger" would have a high matching score. 12% of the U.S. advertisers had a clear match. The key benefit of having the matched advertisers is the ability to measure the direct substitution behavior of these firms.

We also obtained proprietary TikTok data aggregated at the country-day level for all 33 countries in our study, covering the period from January 12 to 26. For the universe of advertisers in each country, the data include total ad spend (USD). Spend is further disaggregated by small and large businesses, based on TikTok's internal classification, which depends on employee number, turnover, or asset size.

2. Extended Methods

2.1. Difference-in-differences model. To assess the impact of the temporary TikTok outage, we employ a difference-in-differences approach, comparing advertiser-level outcomes for ads targeting the U.S. (treated market) to those in other countries (control group), before and on the day of the outage.

Specifically, we estimate the following model:

$$y_{i,c,d} = \beta\ US_{i,c} \times TikTok\ Outage_d + \gamma \mathbf{x}_i \times TikTok\ Outage_d + \delta_i + \lambda_c + \theta_d + \epsilon_{i,c,d} \quad [1]$$

where $y_{i,c,d}$ denotes the outcome variable for advertiser i running ads in country c on day d. The treatment indicator is US, which takes the value 1 when the advertiser runs ads in the US and 0 otherwise. The variable *TikTok Outage* equals 1 on Sunday, January 19 since that is the day TikTok was mostly unavailable and 0 otherwise.

We include several fixed effects and controls. δ_i represents advertiser fixed effects,[¶] which help to account for time-invariant unobservable factors that may be simultaneously correlated with the treatment and the outcomes. λ_c are country fixed effects, which control for constant country-specific characteristics possibly correlated with the outcomes. θ_d are day fixed effects, which control for common time trends across all advertisers. To account for heterogeneous time trends that may affect the treatment and control groups differently, when available, we interact pre-determined advertiser characteristics $\mathbf{x}_i$ with *TikTok Outage*. Depending on the data, these controls can include advertisers' total ad spend in the two months prior to the outage, ad category, USD-currency, and language indicators. We test the sensitivity of our estimates to the inclusion of different controls (e.g., audience size and age), as well as to alternative specifications of fixed effects.

We estimate equation Eq. (1) using OLS, clustering the standard errors at the country level to account for serial and cross-sectional correlation in the errors for all advertisers in the same country. In further analysis, we find that the results hold when we use country-level wild-cluster bootstrapping to estimate the standard errors. Our parameter of interest is β. In order for β to capture the causal effect of the outage on y, the parallel-trends assumption must hold. This means that outcomes on the day of the outage of advertisers with ads in the U.S. and in other countries should have been similar in the absence of the outage. In Table 1 columns (1-8), the panel data is balanced. Table 1 columns (9-10) only focus on days in which advertisers have active campaigns; therefore, the panel is not balanced for these two regressions.

2.2. Event-study model. Although the parallel-trends assumption cannot be directly tested, we provide supporting evidence by estimating the following flexible event-study specification using OLS:

$$y_{i,c,d} = \sum_{d \neq Jan18} \beta_d \left[US_{i,c} \times \theta_d\right] + \gamma \, \mathbf{x}_i \times Post\ Outage_d + \delta_i + \lambda_c + \theta_d + \epsilon_{i,c,d} \qquad [2]$$

where parameters β_d estimate the differences in the outcomes between U.S. and non-U.S. advertisers on different days—one week before and after the outage—compared to the omitted day (Jan 18). The variable *Post Outage* equals 1 on the day of the outage and the subsequent week (Jan 19-26). We cluster the standard errors at the country level. This specification allows us to test whether pre-outage outcomes across treatment and control groups were significantly different, providing evidence corroborating the identifying assumption. Moreover, this model allows us to study the dynamic effects of the outage over the following days. Finally, this analysis, together with a series of placebo exercises, helps to disentangle the effects of the outage from other events occurring near the same time, such as the U.S. presidential inauguration, that could confound our estimates.

2.3. Evidence supporting the identifying assumption. We estimate the event-study model described in Eq. (2) for all outcomes using the SIEP data: spend, CPM, impressions, advertising activity, and ad volume. Figure 1 panels (a) and (b) in the paper show the advertiser spend and CPM results. The results provide support for the parallel-trends assumption: in the days leading up to the TikTok outage, outcomes for ads in the U.S. and other countries are largely comparable. The only exception is impressions, for which we observe a significant pre-trend increase in the U.S. relative to other countries.

We observe wider confidence bands in Figures 1(a), 1(b) and 2(a) during and after the outage compared to the previous days. This variance increase is not mechanically driven by a smaller number of advertisers during or after the outage. Specifically, for Figures 1(a) and 2(a), the panel is balanced. For Figure 1(b), the counts of advertiser-country are slightly higher during and after the outage. Therefore, we believe a higher volatility in advertisers' strategies and actions is a likely explanation for the increase in the width of the confidence bands.

Moreover, for the outcomes above, we conduct several placebo tests to assess trend differences between U.S. and non-U.S. advertisers in the two weeks preceding the outage, using leads of the treatment variable. These regressions generally support the identifying assumption: estimated differences are either small and insignificant or, if significant, they are in the opposite direction of our main effects. As before, the only exception is impressions, for which we observe only a marginally significant increase the day before the outage.

2.4. Potential confounding from the U.S. presidential inauguration for SIEP ads. One potential threat to our identification strategy for the SIEP ads is the presidential inauguration on January 20, 2025. In addition to our finding in the paper that the estimates for launch likelihood and ad count using the non-SIEP data are similar to those for the SIEP data, there are several other reasons to believe that the inauguration is unlikely to have contributed to the increased demand observed on January 19 for the SIEP data. First, across all outcomes, we do not observe any jump in the event-study graphs on the day of the inauguration (Jan 20) relative to the day before. Second, we formally test this by looking at two-day windows around the outage and the inauguration. We compare the day of the outage (Jan 19) with the day before (using the actual treatment indicator), and the day of the inauguration (Jan 20) with the day before (using lags of the treatment variable). Consistent with the event-study graphs, we observe a large and statistically significant increase in the probability of advertising and spend on the day of the outage (3.3 percentage points (p.p.) and 12.3%, respectively; $p < 0.05$) but only a negligible and insignificant change on the day of the inauguration (-0.1 p.p. and -0.4%, respectively). If the inauguration were driving the spike in demand,

[¶] Advertisers are defined by page ID-currency combinations. An alternative specification would be to define advertisers by page ID-currency-country combinations and remove country fixed effects, or to use page ID and country fixed effects, removing currency dummies. We find that the results are quantitatively the same with these alternative specifications.

we would expect the change to occur on that day and not the day before. Even if advertisers wanted to advertise ahead of the inauguration, we would not expect all of it to be concentrated on January 19. Third, we conduct the same two-day analysis using data surrounding the 2021 U.S. presidential inauguration. We do not observe any significant increase in advertising the day before the inauguration (Jan 19) or the day of (Jan 20). These patterns provide supporting evidence that it was the outage and not the inauguration that drove the jump in demand.

2.5. Effects across platforms. Relative to Facebook, Instagram is considered a closer substitute for TikTok. Therefore, we expect the TikTok outage to have had a smaller effect on ads targeting only Facebook users. Indeed, we find that spending on ads targeting only Facebook did not change significantly, while spending on ads targeting only Instagram grew by 3.4% ($p < 0.05$) and spending on ads targeting both Facebook and Instagram grew by 17.8% ($p < 0.05$).

2.6. Reactions of advertisers after the TikTok outage by size. To study the reactions of advertisers to the reintroduction of TikTok, we restrict our attention to advertisers that ran ads on Meta platforms the day of the outage (Sunday, January 19). For them, we estimate another difference-in-differences model, similar to equations Eq. (1) and Eq. (2), where the day of the outage becomes our baseline period, and look at the effects on the subsequent 7 days. We split the sample into larger and smaller advertisers, according to the median advertising spend by country in the two months before the outage.

For smaller advertisers, spend on U.S. ads decreased by 25% the week after the outage, with respect to the day of the outage. The drop in spend was particularly large among smaller advertisers that ran campaigns on Instagram only (-123%) and for those targeting younger demographics on Meta platforms (-60%). All estimates are significant at the 0.01-level. For smaller advertisers running ads on Facebook and those targeting older groups, the reduction in ad spend was much less and not statistically significant. Finally, for larger advertisers, we observe only a 19% reduction in ad spend for those targeting younger demographics, significant at the 0.01-level. In all other cases, estimates are insignificant.

2.7. Robustness to different model specifications. We find that our results are robust to alternative estimators, model specifications, and clustering levels. Specifically, we use the Logit model for binary outcomes and find that the results remain qualitatively unchanged. We also replicate the main analyses using an alternative set of control variables (including country-specific time trends, total past potential audience size and age) and fixed effects (advertiser-country indicators), and find consistent results. Finally, using country-level wild-cluster bootstrap standard errors does not affect the statistical significance of our results (1).

2.8. Robustness to different distribution assumptions on spend and impressions. In the paper, we assume spend and impressions are distributed uniformly over time for the SIEP ads. In this section, we try different distribution assumptions to understand how consequential the uniform distribution assumption is for the results. First, note that when ads are still active (i.e., running during the time of data collection) they do not have an end date: for them, we assume the end date to be the most recent day the data was collected (Feb 14, 2025). Then, we assess the robustness to the following alternative distributions:

1. **Front-load (decreasing linear) impressions and spend:** This distribution captures the common scenario that ad delivery becomes slower as advertisers approach audience saturation. Specifically, total spend and impressions are larger at the beginning, when ads are shown to the most relevant individuals, and as the pool of relevant users dwindles over time, so does the number of impressions and spend.

   Let T represent the number of days an ad is active. Let Q represent the total number of impressions or spend of an ad. For each active day t, we allocate:

   $$\text{Impressions or Spend}_t = Q \cdot \frac{2(T-t+1)}{T(T+1)}.$$

   If $T = 5$, 1/3 of impressions and spend would be allocated to day 1, 4/15 to day 2, etc.

2. **Normally distributed impressions and spend:** This distribution captures the idea that advertisers may use some time to learn to whom they should show their ads. They only increase spend after some initial learning, which could also lead to more impressions. After some time, as the pool of relevant individuals decreases, so too do the spend and impressions.

   Let us assume the impressions and spend are normally distributed with:

   - Mean $\mu = \frac{T+1}{2}$ (centered in the middle of the period)
   - Standard deviation $\sigma = T/4$
   - $\text{Impressions or Spend}_t = Q \cdot \frac{\exp\left(-\frac{(t-\mu)^2}{2\sigma^2}\right)}{\sum_{k=1}^{T} \exp\left(-\frac{(k-\mu)^2}{2\sigma^2}\right)}.$

Finally, for each of the above methods, we calculate the CPM applying the usual formula ($Spend \times 1000/Impressions$). The results are robust to these different assumptions. Overall spend increases by 22.8% ($p < .01$) when we assume spend is front-loaded and by 23.3% ($p < .01$) when we assume spend is normally distributed. For active ads (i.e., those with positive spend and impressions), when impressions and spend are front-loaded, the change in impressions is not statistically significant and CPM increases by 11.3% ($p < .05$). When we assume impressions and spend are normally distributed, the change in impressions is not statistically significant and CPM increases by 13.5% ($p < .05$).

References

1. AC Cameron, JB Gelbach, DL Miller, Bootstrap-based improvements for inference with clustered errors. *The Rev. Econ. Stat.* **90**, 414–427 (2008).
